# Influence of Information Sources and Group Norms on University Students’ Online Rumor Refuting Behavior During Public Health Emergencies

**DOI:** 10.3390/bs15050635

**Published:** 2025-05-07

**Authors:** Hongmei Xia, Zitong Xing, Yu Liu

**Affiliations:** College of Public Administration and Law, Hunan Agricultural University, Changsha 410128, China; 17373635576@stu.hunau.edu.cn (Z.X.); liuyu123@stu.hunau.edu.cn (Y.L.)

**Keywords:** SOR theory, university students, information sources, group norms, online rumor refuting

## Abstract

In the digital era, social media proliferation accelerates rumor dissemination. During public health emergencies, such misinformation intensifies social harm. Studying the influencing factors of online rumor refutation behavior thus becomes crucial. This study uses the stimulus–organism–response (SOR) theory as an analysis framework, based on the perspective of information sources and integrating group norms as a moderating factor, to explore the psychological processes affecting Chinese university students’ online rumor refuting in public health emergencies. Against the backdrop of the COVID-19 pandemic, a questionnaire survey was conducted on 1017 respondents, and the collected data were analyzed using the structural equation modeling research method. The results indicate that both online and offline information seeking positively influence university students’ fear of contracting the COVID-19 virus. University students’ fear positively influences their engagement in online rumor refuting. Notably, fear mediates the link between online and offline information seeking and online rumor refuting. Additionally, group norms help strengthen the connection between university students’ fear and their involvement in online refuting rumors. These results provide theoretical explanations and practical guidance for university students to refute rumors online.

## 1. Introduction

The COVID-19 pandemic four years ago triggered a serious public health crisis that led to a surge in misinformation on social media (Guitton, 2020). Misinformation can adversely affect an individual’s health and exacerbate anxiety, especially among university students (Cao et al., 2020). Active involvement in combating rumors is especially important for socially active students, helping to build a more resilient society. Therefore, studying university students’ online rumor refuting behavior during public health emergencies holds significant theoretical and practical importance.

Research indicates that studies on rumor response behavior mainly focus on two aspects: factors influencing individual rumor transmission and factors affecting the effectiveness of rumor refutation. For example, Liu et al. (2023) explored variables affecting the individual denial and spread of rumors, considering the moderating role of critical thinking. Luo et al. (2021) examined the interaction between peer status, peer communication, fear, and personal rumor sharing, alongside the moderating role of health self-efficacy. These studies applied the SOR theory, primarily focusing on individual-level moderating factors, such as critical thinking (the capacity to interpret information through evidence-based reasoning overcoming cognitive biases) (Liu et al., 2023) and health self-efficacy (individuals’ beliefs in their ability to manage their health) (Luo et al., 2021). However, they overlooked the impact of social group factors on individual online rumor refuting behavior, such as the role of group norms. Group norms, defined as collective behaviors shaped by social identity (Hogg et al., 2002), remain underexplored in their regulatory role over fear-driven rumor refuting behavior (Xia et al., 2024). Additionally, although previous research acknowledges information sources as stimuli, there is relatively little research on the impact of online and offline information seeking on fear and the subsequent behavioral responses (Liu et al., 2023).

Therefore, in the context of the COVID-19 pandemic, this study adopts the stimulus–organism–response (SOR) theory, based on the perspective of information sources, and takes group norms as the moderating factor to explore the psychological processes affecting Chinese university students’ online rumor refuting behavior in the case of public health emergencies. Specifically, the following two questions are focused on: How do online and offline information seeking act as stimuli to trigger fear (organism) and online rumor refuting behavior (response)? To what extent do group norms moderate the relationship between university students’ fear of contracting the COVID-19 virus and online rumor refutation behavior?

## 2. Theoretical Grounding and Hypothesis Development

### 2.1. SOR Theory

The stimulus–organism–response (SOR) theory posits that specific environmental stimuli can influence the emotional and cognitive states of individuals, thereby affecting particular behavioral outcomes (Mehrabian & Russell, 1974). The stimulus refers to external factors influencing an individual’s state, while the organism encompasses the cognitive and emotional states that link stimuli to individual reactions. Responses represent the final behaviors and results exhibited in response to the stimulus.

Notably, previous studies have demonstrated the value of the SOR theory for studying human behavior during the COVID-19 pandemic (Soroya et al., 2021). For example, Yang et al. (2021) found that university students generally resorted to online learning during the COVID-19 epidemic and applied the interest-based SOR theory to examine the online learning participation of Chinese university students. Zheng et al. (2020) employed the SOR theory to explain how citizens can assess the pandemic in terms of psychological distance and why lockdown can alleviate the social anxiety caused by COVID-19. In summary, the SOR theory has frequently been utilized as a framework for studying public online network behavior during the COVID-19 pandemic.

Therefore, this study adopts the SOR theory as an analytical framework to investigate university students’ online rumor refuting behavior. In particular, all types of external information, including online and offline sources, may influence the emotions of university students. This external information can affect the emotional responses of the “organism” (university students), with fear being an important emotional response. Ultimately, these emotional responses will influence students’ behaviors in refuting online rumors. Thus, this study explores the factors influencing university students’ behaviors in refuting online rumors based on the SOR theory, where online or offline information seeking behaviors are used as external stimuli (S), the fear of contracting the COVID-19 virus is used as the internal organism response factor for university students (O), and university students’ behaviors in refuting online rumors are used as the response (R).

### 2.2. Information Seeking and Fear

Information seeking refers to obtaining information through technical means within a defined range for a specific purpose (Agosto & Hughes-Hassell, 2005). Expanding upon current scholarship, this paper categorizes information retrieval into online and offline domains. Various information outlets significantly influence individuals’ emotional responses to the epidemic, as indicated by the research of Zheng (2023). Fear emerges when an individual anticipates adverse consequences or feels out of control in a specific situation. Fear, as defined by Boss et al. (2015), manifests as a response to perceived threats, leading individuals to take protective actions. Within the scope of this investigation, fear specifically denotes the heightened apprehension felt by university students regarding the potential of contracting the COVID-19 virus.

#### 2.2.1. Online Information Seeking and Fear

During infectious disease outbreaks, individuals access relevant information through a variety of channels, especially social media (Kim & Hawkins, 2020). Previous studies typically define online information retrieval as acquiring information via digital social platforms, such as Weibo and Wechat (Liu et al., 2023; Xia et al., 2024). Therefore, this paper defines online information seeking as university students obtaining information about the COVID-19 virus through digital social platforms such as Weibo and WeChat. Superio et al. (2021) found that online information seeking behavior exacerbated university students’ fear of COVID-19. Liu et al. (2023) emphasized the strong positive correlation between fears and both online information seeking and the behavior of rumor refuting. Therefore, online information seeking may also trigger the fear of the COVID-19 virus among university students. Based on these observations, this paper proposes the following hypothesis:

**Hypothesis** **1.** *Online information seeking has a positive impact on university students’ fear of contracting the COVID-19 virus.*

#### 2.2.2. Offline Information Seeking and Fear

Offline information seeking primarily involves obtaining relevant information through face-to-face communication (Liu et al., 2023), such as discussions with classmates, friends, and family, among others. Some scholars also emphasize that offline information seeking refers to acquiring information through traditional print media, such as newspapers and publicity boards (Xia et al., 2024). Therefore, in this paper, offline information seeking includes obtaining information about the COVID-19 virus through traditional print media like newspapers and publicity boards, as well as through discussions with people such as friends and family. Harrigan et al. (2021) found that peer communication can influence individual emotions. Luo et al. (2021) indicated that offline peer communication affects individuals’ fear levels and increases their tendency to spread pandemic-related rumors. Moreover, compared to online information, traditional media has higher credibility and influence (Zhang et al., 2020), making it more likely to impact students’ fear. Thus, offline information seeking may trigger the fear of the COVID-19 virus among university students. Based on these observations, this paper proposes the following hypothesis:

**Hypothesis** **2.** *Offline information seeking has a positive impact on university students’ fear of contracting the COVID-19 virus.*

### 2.3. Fear and Online Rumor Refuting

The feeling of fear will lead to individual protective behaviors to alleviate this fear (Zhang et al., 2020). A review of the previous literature found that when a person encounters rumors about COVID-19 in a public health emergency, fearful people do not seriously think about the rumors but instead make irrational decisions based on false information, including incomplete information or information that is incorrect (Boss et al., 2015). Therefore, in this case, most individuals tend to believe and share the rumors online. However, there are also studies that show that fear not only has an impact on online rumor spreading but also affects the generation of online rumor refuting behavior (Liu et al., 2023). As can be seen from the above, during public health crises, individual fear can potentially lead to both the spread of rumors and the debunking of rumors. A possible reason for this lies in the variations in how different groups respond to fear.

Rumor refuting is the process of exposing a rumor by providing evidence to support the truth (Wen, 2014). In other words, rumor refuting behavior refers to the process in which an individual or a group takes the initiative to correct false information by collecting and disseminating reliable evidence or facts after discovering false information so as to eliminate the misdirection and adverse impact of rumors on the public. This study classifies rumor refuting behaviors into three categories: active and conscious rumor refuting on social platforms, rumor refuting through online interpersonal communication, and passive or unconscious rumor refuting on online platforms. From the perspective of social psychology, the SOR theory can be used to explain the influence of emotional factors on rumor refuting behavior. Different groups exhibit varied responses to rumors, with university students, generally possessing a higher level of education, demonstrating more critical thinking in their cognitive processes. The greater one’s critical thinking abilities, the less likely they are to spread rumors. Additionally, the majority of university students are in their youth, enjoying good physical health and possessing a strong sense of self-efficacy regarding their health. Studies have indicated that high health self-efficacy can weaken the link between fear and spreading rumors (Luo et al., 2021). In summary, university students are more likely to be critical thinkers and have a better sense of their own health self-efficacy. This reduces the likelihood of engaging in rumor dissemination and increases their participation in rumor debunking activities. Therefore, while university students may fear contracting the COVID-19 virus, the emotion of fear more often triggers their behavior in refuting rumors rather than spreading them. Based on these observations, this paper introduces the following hypothesis:

**Hypothesis** **3.** *The fear of contracting the COVID-19 virus positively influences university students’ online rumor refuting behavior.*

### 2.4. Mediating Effects of Fear

On the one hand, based on the SOR theory, this study explored how online and offline information seeking (S) affect rumor refuting behavior (R), with particular attention paid to the mediating role of fear (O). Through online information seeking, individuals can easily access a large number of relevant facts and a lot of evidence, thereby enhancing their ability and inclination to refute rumors. Liang et al. (2022) emphasized that COVID-19-related online information seeking induced individuals’ rumor refuting behaviors. In addition, offline information seeking can enhance individuals’ cognition and judgment ability of rumors through face-to-face communication so as to promote their participation in rumor refuting behavior. Li et al. (2020) emphasized that communication between offline rumor deniers and rumor spreaders can effectively reduce the number of people spreading rumors, curb the spread of rumors, and ultimately play a role in refuting rumors. In summary, both offline and online information seeking directly influence individuals’ online rumor refuting behavior, including that of university students.

On the other hand, existing research indicates that both online and offline information seeking can affect individuals’ emotions, which in turn indirectly influence their behavior. For example, Luo et al. (2021) highlighted the mediating role of the fear of COVID-19 in the relationship between peer communication and online rumor sharing. Liu et al. (2023) noted that online and offline information seeking behavior significantly intensify individuals’ fear, which directly impacts online rumor refuting behavior. This indicates that fear plays a crucial mediating role between online information seeking and rumor refuting behavior. Therefore, online and offline information seeking can not only have a direct impact on university students’ online rumor refuting behavior but also have an indirect impact on university students’ online rumor refuting behavior by affecting their fear of COVID-19. Based on these observations, this paper introduces the following hypotheses:

**Hypothesis** **4.** *The fear of contracting the COVID-19 virus mediated the relationship between online information seeking and online rumor refutation.*

**Hypothesis** **5.** *The fear of contracting the COVID-19 virus mediated the relationship between offline information seeking and online rumor refutation.*

### 2.5. Moderating Effects of Group Norms

As outlined in social identity theory, individuals’ self-perception is molded by their membership in social collectives (Hogg & Abrams, 1988). Furthermore, self-categorization theory asserts that aligning with a distinct social collective fosters the establishment of collective standards, which shape the thoughts and behaviors of members (Hogg et al., 2002). Members within the same group often emphasize discrepancies from individuals outside the group and commonalities among themselves, thereby strengthening their shared identity (Turner et al., 1989). As a result, individuals demonstrate a stronger propensity to conform to behaviors approved by their group (Terry & Hogg, 1996). In China, university education typically follows a class-based teaching model. On the one hand, each student is affiliated with a specific, fixed class. On the other hand, temporary teaching groups are formed based on students’ course selections. The former consists of fixed class members that remain unchanged until graduation, while the latter, temporary teaching groups, vary according to students’ chosen courses. A fixed class functions as a small group, with group behavior within a class unit being prominently shaped by traditional Chinese culture and the emphasis on class cohesion in modern university education. The group norms within these groups wield significant influence.

Existing research indicates that group norms have a significant impact on individual behavior. For example, Gajda et al. (2024) found that group norms play a moderating role between individual psychological characteristics and radical collective action intention. Zhang et al. (2022) found that in the context of an epidemic, individuals pay more attention to the behavior of group members who are physically closer to them, and group norms may profoundly affect individual decision-making. Xia et al. (2024) studied college students’ online rumor refuting behavior during public health emergencies and found that group norms positively moderated the relationship between students’ attitudes toward the virus and their online rumor refuting behavior. In other words, in the group of university students, when most group members participate in online rumor refuting, it can influence others in the group to participate in similar activities. In summary, group norms may positively moderate the relationship between the fear of COVID-19 and online rumor refuting behavior among university students. Based on these observations, this study introduces the following hypothesis:

**Hypothesis** **6.** *Group norms will positively moderate the relationship between university students’ fear and their behavior of online rumor refuting.*

**Figure 1:**
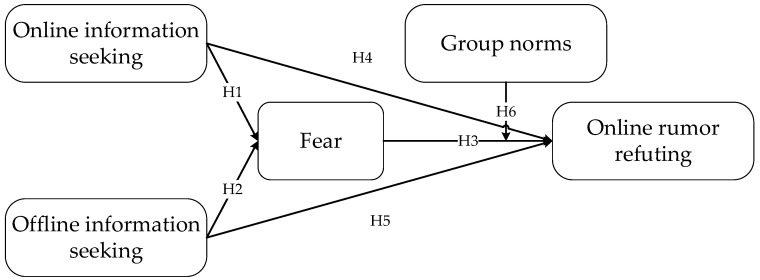
Theoretical model.

This study includes five variables, and the theoretical model is shown in Figure 1.

## 3. Methods

### 3.1. Participants

The research survey, spanning from 8 October 2023 to 15 January 2024, employed a blend of traditional and digital questionnaires for data acquisition. A set of printed questionnaires reached 500 collegiate respondents, while an online questionnaire service, Questionnaire Star (http://www.sojump.com/), was utilized for the virtual segment (Li et al., 2021). In total, 1600 surveys were disseminated via these means, yielding 1315 completed responses. After excluding incomplete questionnaire responses and missing data, 1017 valid responses were left for subsequent analysis. The sample’s demographic information is encapsulated in Table 1, revealing statistics indicating that there were 47.59% male participants, 89.48% aged between 18 and 25, 50.05% studying at junior university levels, and a mere 1.57% sustaining independent lifestyles.

### 3.2. Measures

The survey instrument encompassed dual segments: demographic particulars and study-related constructs. Information regarding gender, age group, educational background, and living condition comprised the initial segment. The subsequent part delved into specific constructs, employing a Likert scale ranging from 1 to 7.

### 3.3. Dependent Variables and Independent Variables

First, in the domain of online information seeking, we began by evaluating participants’ tendencies toward seeking virus-related information online, using three elements that stemmed from Oh et al. (2013). For example, “I frequently turn to platforms like Weibo for virus-related updates during the epidemic”.

Second, for offline information seeking, we assessed participants’ inclinations using three elements adapted from Suki and Suki (2019). An example includes, “I often converse about virus-related topics with peers and family members during times of crisis”.

Third, regarding fear assessment, we examined participants’ perspectives utilizing a set of three elements that stemmed from Boss et al. (2015) to capture university students’ perceptions of pandemic-related information. An example from this assessment reads, “I worried about the possibility of infection from others”.

Fourth, moving on to group norms, we employed a scale incorporating three elements modified from White et al. (2008) and Robinson et al. (2016). An example includes, “Many of my acquaintances have been actively involved in dispelling rumors amidst the ongoing pandemic”.

Lastly, when analyzing online rumor refuting, we evaluated participants’ behaviors in debunking falsehoods using three elements drawn from Zhang et al. (2021) and Liu et al. (2023). An example statement is, “I have disseminated factual information to counter epidemic-related rumors on platforms such as Weibo during the outbreak”.

### 3.4. Covariates

Additional variables known as covariates, extending beyond independent variables, have the potential to exert an impact on the results of a study. Studies have indicated that demographic aspects like gender, age group, educational background, and living condition have the capacity to shape how individuals perceive and act (Luo et al., 2021). Thus, to account for these influences, we integrated these factors into our research model.

## 4. Data Analysis

### 4.1. Measurement Model

Prior to commencing the regression analysis, the constructs’ reliability and validity were evaluated through the utilization of AMOS 27.0 software. To assess construct validity, the researchers employed confirmatory factor analysis, adhering to the guidelines proposed by Marsh et al. (2004). The outcomes illustrated a favorable alignment with the data (χ^2^/df = 3.540; CFI = 0.978; IFI = 0.978; TLI = 0.971; RMSEA = 0.050; PNFI = 0.739; PCFI = 0.745).

The initial assessment predominantly concentrated on assessing convergent validity, as outlined in Table 2, where all item loadings exceeded the threshold of 0.7, duly meeting the criteria. Additionally, the values for Cronbach’s alpha surpassed 0.7, denoting a robust internal consistency. Composite reliabilities (CR) for all constructs were observed to be higher than 0.7, affirming their reliability. Furthermore, AVE values surpassing 0.6 served to reinforce the findings related to convergent validity, in line with earlier research by Fornell and Larcker (1981).

In addition, the discriminant validity of the constructs is illustrated in Table 3. All square roots of AVE proved superior to the respective correlation coefficients, ensuring sufficient discriminant validity. Subsequently, for the evaluation of multicollinearity, the variance inflation factor (VIF) was computed. The findings showcased VIF values spanning from 1.061 to 1.495, with tolerances surpassing 0.1, thus indicating the absence of notable multicollinearity concerns (Zhang et al., 2021). Finally, to address common method bias (CMB), Harman’s single-factor test was employed. The execution of exploratory factor analysis revealed a primary factor loading of 33.428%, falling below the suggested threshold of 50% (Podsakoff et al., 2012), implying minimal CMB influence within our study.

### 4.2. Structural Model

The goodness-of-fit indices of the theoretical framework were evaluated through the structural model. The adjusted model demonstrated a strong fit, supported by the following indices: χ^2^/df = 3.589; CFI = 0.982; TLI = 0.976; PNFI = 0.710; PCFI = 0.714; and RMSEA = 0.050. We utilized SPSS to estimate the regression coefficients, with the results detailed in Table 4. In Model 1, both online information seeking and offline information seeking were positively and significantly linked to fear (β = 0.101, *p* < 0.05 and β = 0.096, *p* < 0.01, respectively). The above results support H1 and H2. Moving to Model 2, a strong positive correlation was found between fear and online rumor refuting. The fear coefficient was both positive and statistically significant (β = 0.263, *p* < 0.001), indicating a notable impact of fear on individuals’ online rumor refuting behavior and confirming support for H3.

### 4.3. Moderated Effect Analysis

Subsequently, we examined the moderation effects of group norms (H6). This study employed SPSS 27.0 software to perform hierarchical regression analysis on the data, aiming to empirically verify the moderating effects. The detailed results are presented in Table 4. In Model 4, group norms were found to significantly enhance the relationship between fear and individuals’ online rumor refuting (FR×GN: β = 0.039, *p* < 0.05). This indicated that when individuals adhered to higher group norms, the positive effects of fear on online rumor refuting were notably amplified. These findings reinforce the support for H6.

### 4.4. Mediated Effect Analysis

We examined the mediation effects of fear as outlined by Luo et al. (2018) through a multi-step process, with the results summarized in Table 5. Initially, in Model 5, we assessed the impact of online information seeking and offline information seeking on online rumor refuting behavior, observing positive and significant effects (ONIS: β = 0.112, *p* < 0.01; OFFIS: β = 0.149, *p* < 0.001). Subsequently, in Model 6, we investigated the influence of the mediator, fear, on online rumor refuting behavior, revealing positive effects (β = 0.263, *p* < 0.001). Moving on to Model 7, we explored the relationships between online information seeking, offline information seeking, and fear, uncovering significant and positive associations (ONIS: β = 0.101, *p* < 0.05; OFFIS: β = 0.096, *p* < 0.01). Finally, in Model 8, we combined online information seeking, offline information seeking, and fear in a regression analysis to assess their collective impact on online rumor refuting behavior. The coefficients for all three variables were significant and positive. The coefficients for online information seeking and offline information seeking were comparatively smaller than those in the initial steps (ONIS: 0.089 < 0.112; OFFIS: 0.127 < 0.149). This indicates that fear served as a partial mediator in the relationships between online information seeking, offline information seeking, and online rumor refuting behavior, thereby confirming support for H4 and H5.

In summary, Hypothesis 1 is supported, indicating that online information seeking positively affects university students’ fear of COVID-19 infection. Similarly, Hypothesis 2 is validated, suggesting that offline information seeking also positively affects their fear of infection. Hypothesis 3 is supported, showing that the fear of COVID-19 infection positively influences university students’ online rumor refuting behavior. Additionally, Hypotheses 4 and 5 are confirmed, demonstrating that fear mediates the relationship between both online and offline information seeking and online rumor refuting behavior. Finally, Hypothesis 6 is supported, indicating that group norms positively moderate the relationship between fear and online rumor refuting behavior.

## 5. Discussion and Implications

### 5.1. Discussion

Firstly, in accordance with the first and second hypotheses, both online and offline information seeking had positive effects on university students’ fear of contracting the COVID-19 virus. Specifically, online information seeking through social media platforms such as Weibo and Twitter, information seeking by having discussions with friends and family to acquire relevant knowledge, and offline information seeking through traditional media channels all have a positive impact on university students’ fear of contracting the COVID-19 virus. The conclusions of this study are consistent with the findings of Liu et al. (2023), who emphasized that online information search and offline peer communication can trigger individuals’ fear of the COVID-19 virus. At the same time, Wang and Zhuang (2018) pointed out that although social media is widely used, traditional media remains an important and trusted source of reliable information, particularly during unforeseen events. In other words, different information sources affect personal emotions. Especially during uncertain events, offline traditional media plays a crucial role. The expanding roles of traditional media in China, alongside offline societal interactions, still significantly impact personal information decisions, particularly within the Chinese context (Huang & Lu, 2017). It is worth noting that the information obtained through school bulletin boards, teacher–student exchanges, and offline peer interactions greatly influenced university students’ fear of contracting the COVID-19 virus.

Secondly, when the third hypothesis was considered, it was realized that the fear of contracting the COVID-19 virus positively influences university students’ online rumor refuting behavior. When university students’ fear of contracting the COVID-19 virus is stronger, the possibility of participating in rumor refuting behavior will increase. This outcome aligns with the research of Liu et al. (2023), which also suggests a positive relationship between personal fear and endeavors to counter rumors. However, contrary studies have proposed a positive link between fear and rumor dissemination (Luo et al., 2021), conflicting with this study’s findings. Essentially, prior studies have suggested that fear can prompt both the propagation and debunking of online rumors. Notably, these investigations did not address variances among distinct cohorts, while this study predominantly scrutinizes the online rumor refuting tendencies of university students. University students usually exhibit certain characteristics, such as better perceived physical health. And they demonstrate high levels of media literacy, especially in critical thinking and cognition. Previous research has indicated that qualities such as health self-efficacy, critical thinking, and cognitive abilities can moderate the relationship between fear and online rumor refuting behavior among university students (Liu et al., 2023; Luo et al., 2021). Additionally, Wang et al. (2022) emphasized that cognitive capabilities impact individuals’ ability to judge rumors. Higher personal cognitive abilities enhance rumor discrimination, reducing the likelihood of rumor dissemination and increasing the probability of online rumor refuting. As a result, university students will rationally choose to refute rumors instead of spreading rumors when they are afraid of viruses, which makes fear and online refuting behaviors show a positive correlation.

Thirdly, considering the fourth and fifth hypotheses, fear played a partial mediating role in the relationship between online information seeking, offline information seeking, and online rumor refuting behavior. On the one hand, online information seeking can influence university students’ online rumor refuting behaviors through fear. An essential aspect of searching for information online, particularly on social media platforms, is the exposure to diverse viewpoints. This exposure may lead users to encounter rumors and a plethora of additional information, including discussions on popular science topics related to those rumors and relevant knowledge (Wang et al., 2018). Having immediate access to comprehensive information reduces the likelihood of blindly accepting rumors. Consequently, online information seeking may lower the likelihood of rumor propagation and increase the possibility of online rumor refuting. On the other hand, offline information seeking can also affect university students’ online rumor refuting behaviors through fear. Sudden public health crises often trigger anxiety among individuals. To manage this anxiety, people typically turn to traditional offline media for reliable information (Ahsan et al., 2019) or seek guidance from friends and family. Traditional offline media has long been a trusted source of official information. In contrast to the evolving landscape of online social media, traditional media sources are viewed as credible institutions and individuals. The link between credibility and authenticity implies that more credible information is deemed more authentic (Li & Sakamoto, 2014), thereby reducing the likelihood of rumor circulation and facilitating online rumor debunking.

Finally, when the sixth hypothesis was considered, it was found that group norms strengthened the relationship between fear and online rumor refuting. The conclusion of this study is similar to the results of Xia et al. (2024), who found that group norms positively strengthen the relationship between university students’ attitudes and the behavior of online rumor refuting. Previous studies consistently illustrate the substantial predictive power of group norms on conduct. For instance, Robinson et al. (2016) noted that group norms play an important role in predicting sun protection decisions for a group of young Australian women. University students are notably vulnerable to the impact of group norms. In the majority of Chinese universities, a class-based teaching model is employed, where each university student belongs to a specific class, forming a small group. Concurrently, Chinese university education emphasizes fostering class cohesion, meaning that the behaviors of class members exhibit consistency. In other words, if the majority of students in a class engage in online rumor refuting activities, the remaining members of the class are also likely to participate in online rumor refuting. Furthermore, university students are prone to engaging in conformity behaviors within a group setting. Consequently, when a predominant behavior within a group involves debunking rumors online, individuals are inclined to conform and follow the prevailing trend of online rumor refuting. Consequently, group norms are pivotal in shaping the connection between university students’ fear and their actions regarding rumor refutation.

### 5.2. Theoretical Implications

On the one hand, this study expands the theoretical research of SOR. This study applies the SOR theory to investigate online rumor refuting conduct amid public health crises, delineating three pertinent factors: stimulus (comprising online and offline information searches), organism (fear), and response (online rumor refuting behavior). While previous research has recognized fear’s significance as a mediator in IS-related studies (Boss et al., 2015; Sela et al., 2020; Vranjes et al., 2017), limited attention has been given to fear’s mediating function in online rumor refuting behavior. This study found that both online and offline information seeking positively influence fear, which directly impacts online rumor refuting behavior. Moreover, fear serves as a mediator between information seeking and rumor refuting behavior. Therefore, this study essentially clarifies the mechanisms by which different information sources influence university students’ online rumor refuting behavior. This not only enriches the discourse on online rumor refuting practices but also expands the research on the SOR theory.

On the other hand, this research illuminates the influence of group norms on university students’ online rumor refuting conduct amid public health crises. This article introduces group norms from the realm of social psychology as a moderating factor, thereby enhancing the available literature on factors that impact rumor behavior. Group norms have garnered widespread attention across various research domains, influencing outcomes such as individual behaviors and performances (Baker & White, 2010). However, their specific role in university students’ online rumor refuting behaviors during crises remains relatively unexplored. Through incorporating group norms as a moderating variable in our research framework, we uncovered their substantial amplification of the positive association between fear and online rumor refuting behavior. Therefore, this study not only enhances the understanding of online rumor refuting practices but also highlights the pivotal moderating influence of group norms within this context.

### 5.3. Practical Implications

On the one hand, information management in public health emergencies should be strengthened. The government needs to oversee both online and offline information flow, enhance students’ cybersecurity awareness, and reinforce rumor monitoring systems. Schools can establish offline information exchange areas, and social media platforms should strictly review content and promptly refute rumors. Students should respond rationally to the pandemic by obtaining information from authoritative sources and verifying it with teachers.

On the other hand, group norms should be leveraged to guide students in actively participating in online rumor refutation. Educational institutions can encourage schools to involve students in online rumor refutation as a key measure of teaching quality. Schools can use the power of group norms to identify and encourage classes that participate in online rumor refutation, thereby enhancing class unity and regulating student behavior. Homeroom teachers can organize activities to boost skills and cohesion, promoting constructive online rumor refuting behavior and increasing student participation.

## 6. Conclusions and Limitations

### 6.1. Conclusions

The present study aims to determine the impact of information seeking on university students’ rumor refuting behavior and to examine the mediating role of fear in this relationship, as well as the moderating effects of group norms on the relationship between fear and online rumor refuting behavior. The research conclusions of this paper are as follows: Firstly, the results showed that both online and offline information seeking had positive effects on university students’ fear of contracting the COVID-19 virus. Secondly, the results illustrate that university students’ fear of contracting the COVID-19 virus positively influences their engagement in online rumor refuting. Thirdly, the fear of contracting the COVID-19 virus serves as a mediating factor between online information seeking and online rumor refuting, as well as between offline information seeking and online rumor refuting. Finally, group norms positively moderate the relationship between university students’ fear and the behavior of online rumor refuting.

### 6.2. Limitations and Future Research Directions

While this article offers valuable insights into dispelling online rumors, it is crucial to acknowledge specific constraints. This research primarily concentrates on Chinese university students, with variations in debunking practices likely across diverse cultural environments. To overcome this limitation, forthcoming studies could incorporate cross-cultural datasets to conduct a more comprehensive examination. Furthermore, this study solely examines fear as an intermediary variable. Broadening the investigation to encompass additional facets of information interpretation, such as beliefs concerning rumors, trusted information sources, and engagements on social platforms, could prove beneficial. Additionally, this study exclusively regards group norms as a moderating element. Nonetheless, the existing literature indicates that individual decisions regarding rumor clarification may be shaped by personal attributes and contextual factors. Consequently, upcoming research endeavors could further scrutinize these internal and external parameters.

## Figures and Tables

**Table 1:** Descriptive statistics of respondents’ profile (N = 1017).

Characteristics	Levels	Frequency	Percentage (%)
Gender	Male	484	47.59
	Female	533	52.41
Age group	<18	86	8.46
	18–25	910	89.48
	26–30	13	1.28
	>30	8	0.79
Educational background	Junior university students	509	50.05
	Undergraduate	369	36.28
	Postgraduate	139	13.67
Living condition	Live alone	16	1.57
	Live with roommates	931	91.54
	Live with relatives	67	6.59
	Live with others	3	0.29

**Table 2:** Reliability and validity.

	Items	Factor Loading	Cronbach’s Alpha	CR	AVE
Online information seeking (ONIS)	ONIS1	0.865	0.852	0.875	0.702
	ONIS2	0.895			
	ONIS3	0.746			
Offline information seeking (OFFIS)	OFFIS1	0.814	0.860	0.875	0.701
	OFFIS2	0.837			
	OFFIS3	0.860			
Fear (FR)	FR1	0.871	0.886	0.921	0.795
	FR2	0.921			
	FR3	0.882			
Group norms (GN)	GN1	0.873	0.892	0.900	0.751
	GN2	0.906			
	GN3	0.819			
Online rumor refuting (ORR)	ORR1	0.884	0.894	0.902	0.755
	ORR2	0.868			
	ORR3	0.855			

**Table 3:** Correlations and discriminant validity.

	ONIS	OFFIS	FR	GN	ORR
ONIS	**0.838**				
OFFIS	0.601 ***	**0.837**			
FR	0.157 ***	0.170 ***	**0.871**		
GN	0.208 ***	0.298 ***	0.209 ***	**0.867**	
ORR	0.184 ***	0.229 ***	0.257 ***	0.537 ***	**0.869**

Note: The bold diagonal values in italics represent the square root of AVE. *** *p* < 0.001. ONIS = online information seeking; OFFIS = offline information seeking; FR = fear; GN = group norms; ORR = online rumor refuting.

**Table 4:** Estimation results.

Variables	FR		ORR		ORR		ORR	
	Model 1		Model 2		Model 3		Model 4	
	b	SE	b	SE	b	SE	b	SE
ONIS	0.101 *	0.039						
OFFIS	0.096 **	0.037						
FR			0.263 ***	0.033	0.164 ***	0.030	0.168 ***	0.030
GN					0.515 ***	0.034	0.512 ***	0.034
FR×GN							0.039 *	0.019
Constant	4.128 ***	0.432	2.183 ***	0.444	0.668	0.413	0.609	0.414
Control variables	controlled		controlled		controlled		controlled	
F	7.164		22.780		61.327		53.304	
R^2^	0.041		0.101		0.267		0.270	
Adjusted R^2^	0.035		0.097		0.263		0.265	

Note: * *p* < 0.05, ** *p* < 0.01, *** *p* < 0.001. ONIS = online information seeking; OFFIS = offline information seeking; FR = fear; GN = group norms; ORR = online rumor refuting.

**Table 5:** Estimation results of mediation effects of fear.

Variables	ORR		ORR		FR		ORR	
	Model 5		Model 6		Model 7		Model 8	
	b	SE	b	SE	b	SE	b	SE
ONIS	0.112 **	0.042			0.101 *	0.039	0.089 *	0.041
OFFIS	0.149 ***	0.039			0.096 **	0.037	0.127 **	0.038
FR			0.263 ***	0.033			0.231 ***	0.033
Constant	2.181 ***	0.460	2.183 ***	0.444	4.128 ***	0.432	1.228 **	0.469
Control variables	controlled		controlled		controlled		controlled	
F	16.060		22.780		7.164		21.516	
R^2^	0.087		0.101		0.041		0.130	
Adjusted R^2^	0.082		0.097		0.035		0.124	

Note: * *p* < 0.05, ** *p* < 0.01, *** *p* < 0.001. ONIS = online information seeking; OFFIS = offline information seeking; FR = fear; ORR = online rumor refuting.

## Data Availability

The raw data supporting the conclusions of this article will be made available by the authors on request.
